# Medical and Physical Intelligence

**Published:** 1823-11

**Authors:** 


### MEDICAL AND PHYSICAL INTELLIGENCE.

1. *Meteorological Society of London.*—A MEETING was lately held at the London Coffee-house, Ludgate-hill, to take into consideration the propriety of forming a Meteorological Society. Dr. BIRKBECK was in the chair; and it was agreed that the Society shall be conducted by a President, Vice-Presidents, Treasurer, Secretary, and Council; that two guineas be paid annually, in advance, by each member; that no other qualification be required to constitute eligibility to this Society, than a desire to promote the science of meteorology; &c. &c.

---

### PATHOLOGY.

2. *Hissing Noise in the Left Cavity of the Chest.*—MM. ROSSI and FENOGLIO, of Turin, relate the case of a person in whom a hissing noise proceeded from the left side of the chest, and was so loud as to be heard at some feet distant. The man assured them that, when in church, the noise was so great as to attract the notice of those who were near him. He attributed this disease to a fit of passion, which he said had been immediately followed by the hissing. M. Rossi made him strip, and take full inspirations, at the same time examining the principal points by the method of percussion, but without being able to detect any pulmonary lesion, aneurism, or polypus. The diastole of the heart, as well as the beating of the pulse, corresponded to the hissing; they were very strong, whilst the systole was weak. The power which produced the diastole was so constantly active, that the systole was little felt at the radial arteries. M. Rossi thinks that the hissing proceeded from the heart, and gives the following explanation:—1st. Either the blood passing from the left ventricle into the aorta, not only during the diastole, but likewise during the systole, was propelled with great impetuosity; or, 2d, the fluid encountered in its passage into the aorta some resistance, the effort to surmount which occasioned the hissing. Hence he concluded that the cause of this noise was in the orifice of the aorta. The patient died leucophlegmatic some time after: the body was opened, and the valves at the orifice of the aorta were found to be schirrous.—The commentator upon this case, in the Revue Medicale, doubts the accuracy of the explanation offered by Rossi, and conjectures that the hissing noise depended on some peculiar affection of the larynx. *Query:* Is it likely that two medical men, who saw and examined the patient, would refer to the region of the heart a sound proceeding from the larynx, as insinuated by their French commentator. —(*Revue Medicale*, Sept. 1823.)

---

### THERAPEUTICS.

3. *On the Employment of Iron in advanced Stages of Utero-Gestation.**—Mr. HUTCHINSON thinks it necessary to state to the respectable Editors of the London Medical and Physical Journal, that the cases of Neuralgia narrated in the last Number, by Mr. ILIFF, of Lambeth-road, are important ones, and of which Mr. H. thinks himself obliged to Mr. I. for the well-described history. The fortunate termination of the case of Ann Guymer, (in an advanced state of pregnancy,) one of Mr. Iliff's patients, would apparently justify the mode of its treatment. Mr. H. will, however, take the liberty of cautioning practitioners against the use of large doses of the carbonate of iron in neuralgic diseases occurring during a state of utero-gestation, abortion being the most probable effect of so injudicious and hazardous a practice. Mr. Hutchinson is induced to offer this remark and caution to the numerous readers of the London Medical and Physical Journal, in consequence of a lady, in the neighbourhood of Southwell, long afflicted with tic douloureux in the facial nerves, being recommended, by an

* In a letter to the Editors of the Medical and Physical Journal.

eminent physician and a surgeon, to make use of Mr. H.'s plan of treatment with the iron, in the latter month of her pregnancy. The consequence may be readily anticipated—abortion of a dangerous character.

---

### MISCELLANEOUS.

3. *New Question in Legal Medicine.*—We occasionally see children born with one or more supernumerary toes or fingers, with teeth already developed, and other peculiarities of organization, but these seldom pervade whole generations. In one of the German Journals of the present year, we find an account of two families, named Wolter and Gashow, in which all the individuals are born with a sixth finger and toe! These supplementary fingers are only the sixth-part of the size of the ring-finger, and have only one or two phalanges. A child was born in one of these families without the supernumerary finger, and this gave rise to a curious question in legal medicine. The father, who lived on bad terms with his wife, took occasion to commence a process against her, suing for a divorce, on the plea of adultery being proved by the absence of the family-mark. This being the only proof against his wife, he was nonsuited. The boatswain said he w[illegible] not father the child which [illegible] was born with a wooden leg.

---

### OBITUARY.

---

### DR. BAILLIE.

### METEOROLOGICAL JOURNAL,

*From September* 20, *to October* 19, 1823.

By Messrs. William Harris and Co. 50, Holborn, London.

| | Moon. | Rain gauge | Therm. | | | Barom. | | De Luc's Hyg. | | Wind. | | Atmospheric Variation. | | |
|---|---|---|---|---|---|---|---|---|---|---|---|---|---|---|
| | | | 9 A.M. | Max. | Min. | 9 A.M. | 10 P.M. | 9 A.M. | 10 P.M. | 9 A.M. | 10 P.M. | 9 A.M. | 2 P.M. | 10 P.M. |
| Sep. | | | | | | | | | | | | | | |
| 20 | ○ | | 55 | 62 | 46 | 29.92 | 29.86 | 77 | 79 | W | W | Fine | Fine | Fine |
| 21 | | | 57 | 61 | 50 | 29.70 | 29.35 | 75 | 85 | SSW | SW | Cloud. | Rain | Rain |
| 22 | | | 53 | 55 | 47 | 29.16 | 29.35 | 80 | 84 | SSW | WNW | Cloud. | Sho'ry | |
| 23 | | | 49 | 54 | 44 | 29.74 | 29.24 | 80 | 90 | NW | SW | Fine | Fine | |
| 24 | | | 56 | 66 | 44 | 29.66 | 29.74 | 90 | 91 | SW | SW | Rain | Fine | Fine |
| 25 | | .52 | 57 | 60 | 45 | 29.81 | 29.80 | 87 | 85 | SSW | S | Cloud. | | |
| 26 | | | 57 | 65 | 47 | 29.66 | 29.60 | 84 | 80 | SSW | SW | Fine | | Cloud. |
| 27 | ☾ | | 50 | 65 | 40 | 29.60 | 29.62 | 79 | 81 | NW. | W | | | Fine |
| 28 | | | 46 | 60 | 40 | 29.70 | 29.80 | 78 | 76 | W | NE | Foggy | | |
| 29 | | | 45 | 54 | 40 | 29.80 | 29.66 | 80 | 84 | NE | ENE | Fine | | |
| 30 | | | 45 | 54 | 42 | 29.24 | 29.14 | 80 | 85 | SSE | WSW | Rain | Rain | Rain |
| Oct. | | | | | | | | | | | | | | |
| 1 | | .27 | 50 | 44 | 38 | 28.55 | 29.00 | 95 | 90 | SSE | WSW | | | |
| 2 | | | 46 | 50 | 41 | 29.22 | 29.40 | 85 | 80 | SW | NW | Fine | Fine | Fine |
| 3 | | | 44 | 51 | 45 | 29.65 | 29.70 | 74 | 76 | WSW | S | | | |
| 4 | ● | | 47 | 56 | 45 | 29.80 | 29.91 | 71 | 74 | WNW | WNW | | | Foggy |
| 5 | | | 53 | 58 | 45 | 29.87 | 29.76 | 70 | 75 | SE | SE | Cloud. | Fine | Cloud. |
| 6 | | | 60 | 57 | 45 | 29.67 | 29.64 | 80 | 85 | SE | SE | Fine | Rain | Cloud. |
| 7 | | | 55 | 59 | 43 | 29.75 | 29.97 | 80 | 77 | SSW | WSW | | Fine | Fine |
| 8 | | | 45 | 60 | 46 | 29.97 | 29.86 | 74 | 76 | SW | SSW | | | |
| 9 | | | 50 | 54 | 43 | 29.44 | 29.48 | 85 | 80 | SW | SW | Rain | | |
| 10 | | | 46 | 50 | 47 | 29.34 | 29.25 | 76 | 70 | S | S | Fine | Sho'ry | |
| 11 | | .15 | 45 | 54 | 48 | 28.53 | 29.07 | 83 | 87 | S | S | Storm. | Fine | Cloud. |
| 12 | ☽ | | 43 | 54 | 45 | 29.10 | 29.20 | 84 | 85 | SSW | NE | Rain | | Fine |
| 13 | | | 43 | 51 | 44 | 29.13 | 29.24 | 83 | 80 | NE | SSE | Fair | Rain | Cloud. |
| 14 | | | 41 | 48 | 40 | 29.35 | 29.35 | 85 | 80 | ESE | WSW | Foggy | Sho'ry | |
| 15 | | | 40 | 46 | 40 | 29.34 | 29.34 | 80 | 81 | SW | SSW | | Fine | Fine |
| 16 | | | 41 | 47 | 39 | 29.46 | 29.53 | 84 | 80 | SSW | SW | Fine | | |
| 17 | | | 40 | 47 | 40 | 29.50 | 29.48 | 80 | 78 | SW | SW | | | |
| 18 | | | 41 | 46 | 45 | 29.35 | 29.40 | 89 | 85 | E | SSE | Rain | Rain | Cloud |
| 19 | ○ | .15 | 54 | 57 | 50 | 29.55 | 29.70 | 88 | 90 | E | ENE | | | |

The quantity of rain fallen in the month of September, was 62.100ths.

